# Squamosamide derivative FLZ protects dopaminergic neurons against inflammation-mediated neurodegeneration through the inhibition of NADPH oxidase activity

**DOI:** 10.1186/1742-2094-5-21

**Published:** 2008-05-28

**Authors:** Dan Zhang, Xiaoming Hu, Sung-Jen Wei, Jie Liu, Huiming Gao, Li Qian, Belinda Wilson, Gengtao Liu, Jau-Shyong Hong

**Affiliations:** 1 Laboratory of Pharmacology and Chemistry, National Institute of Environmental Health Sciences, National Institutes of Health, Research Triangle Park, NC 27709, USA; 2 Laboratory of Molecular Toxicology, National Institute of Environmental Health Sciences, National Institutes of Health, Research Triangle Park, NC 27709, USA; 3 Inorganic Carcinogenesis, Laboratory of Comparative Carcinogenesis, NCI at NIEHS, Research Triangle Park, North Carolina 27709, USA; 4 Comprehensive Center for Inflammatory Disorders, University of North Carolina, Chapel Hill, NC 27599, USA; 5 Department of Pharmacology, Institute of Materia Medica, Chinese Academy of Medical Sciences & Peking Union Medical College, 1 Xian Nong Tan Street, Beijing 100050, ProC

## Abstract

**Background:**

Inflammation plays an important role in the pathogenesis of Parkinson's disease (PD) through over-activation of microglia, which consequently causes the excessive production of proinflammatory and neurotoxic factors, and impacts surrounding neurons and eventually induces neurodegeneration. Hence, prevention of microglial over-activation has been shown to be a prime target for the development of therapeutic agents for inflammation-mediated neurodegenerative diseases.

**Methods:**

For *in vitro *studies, mesencephalic neuron-glia cultures and reconstituted cultures were used to investigate the molecular mechanism by which FLZ, a squamosamide derivative, mediates anti-inflammatory and neuroprotective effects in both lipopolysaccharide-(LPS)- and 1-methyl-4-phenylpyridinium-(MPP^+^)-mediated models of PD. For *in vivo *studies, a 1-methyl-4-phenyl-1, 2, 3, 6-tetrahydropyridine-(MPTP-) induced PD mouse model was used.

**Results:**

FLZ showed potent efficacy in protecting dopaminergic (DA) neurons against LPS-induced neurotoxicity, as shown in rat and mouse primary mesencephalic neuronal-glial cultures by DA uptake and tyrosine hydroxylase (TH) immunohistochemical results. The neuroprotective effect of FLZ was attributed to a reduction in LPS-induced microglial production of proinflammatory factors such as superoxide, tumor necrosis factor-α (TNF-α), nitric oxide (NO) and prostaglandin E_2 _(PGE_2_). Mechanistic studies revealed that the anti-inflammatory properties of FLZ were mediated through inhibition of NADPH oxidase (PHOX), the key microglial superoxide-producing enzyme. A critical role for PHOX in FLZ-elicited neuroprotection was further supported by the findings that 1) FLZ's protective effect was reduced in cultures from PHOX^-/- ^mice, and 2) FLZ inhibited LPS-induced translocation of the cytosolic subunit of p47^PHOX ^to the membrane and thus inhibited the activation of PHOX. The neuroprotective effect of FLZ demonstrated in primary neuronal-glial cultures was further substantiated by an *in vivo *study, which showed that FLZ significantly protected against MPTP-induced DA neuronal loss, microglial activation and behavioral changes.

**Conclusion:**

Taken together, our results clearly demonstrate that FLZ is effective in protecting against LPS- and MPTP-induced neurotoxicity, and the mechanism of this protection appears to be due, at least in part, to inhibition of PHOX activity and to prevention of microglial activation.

## Background

Accumulating evidence indicates that chronic inflammation plays a critical role in the pathogenesis of many neurodegenerative diseases such as Alzheimer's disease (AD) [1], Parkinson's disease (PD) [2], multiple sclerosis [3] and macular degeneration [4]. Inflammation in the brain is characterized by over-activation of microglia [5], the resident immune cells in the central nervous system, resulting in dysregulated inflammatory processes. Activation of microglia in the substantia nigra (SN) of PD patients was first reported in 1988 [6]. The presence of activated microglia in the SN has also been observed in animal models of PD [7], which include direct administration of the inflammagen lipopolysaccharide (LPS) into the brain [8]. Inhibition of the glial over-reaction and the inflammatory processes may thus represent a prime target for the development of novel therapeutic agents for these neurodegenerative diseases.

**Figure 1:**
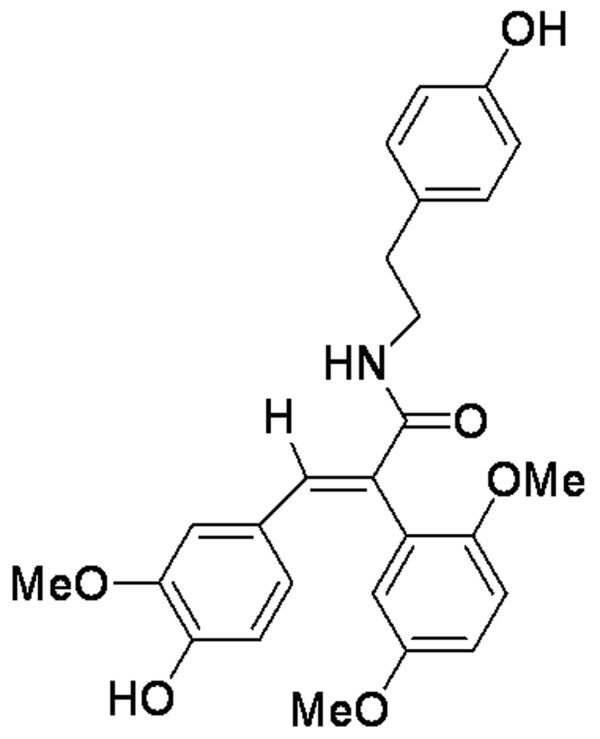
The chemical structure of FLZ.

*Annona glabra *is a tropical fruit tree, the leaf and root of which have been used as traditional Chinese medicines. Recently, several effective components have been isolated from *Annona glabra *and found to have biological activities such as anti-cancer and anti-apoptosis effects [9,10]. Among these, a natural squamosamide from *annona glabra *has been isolated and found to have anti-oxidant activity [11]. After chemical structure modification, a cyclic analogue of this squamosamide with stronger anti-oxidant activity was synthesized as N-[2-(4-hydroxy-phenyl)-ethyl]-2-(2, 5-dimethoxy-phenyl)-3-(3-methoxy-4-hydroxy-phenyl)-acrylamide, and this novel synthetic compound was named FLZ (Fig. 1). We have previously shown that FLZ has protective effects against neuronal damage and neuronal death induced by hydrogen peroxide, glutamate, N-methyl-d-asparatate, hemoglobin and ischemia-reoxygenation [12-14]. Although our previous reports demonstrated the neuroprotective effect of FLZ, the role of microglia in this FLZ-elicited neuroprotection and the molecular mechanisms underlying the protective actions of FLZ were not studied. The purpose of this study was to delineate the role of inflammation in FLZ-induced neuroprotection.

In this study, LPS-induced degeneration of dopaminergic (DA) neurons in mesencephalic neuronal-glial cultures was used as an *in vitro *model for studying the role of microglia in the therapeutic effect of FLZ [15-17]. In this model, LPS directly induces microglial over-activation with subsequent release of a large amount of proinflammatory factors that damage neurons [15]. Here we show that the neuroprotective effect of FLZ is attributable to a reduction in LPS-induced microglial production of proinflammatory factors, such as superoxide, tumor necrosis factor-α (TNF-α), nitric oxide (NO) and prostaglandin E_2 _(PGE_2_). Mechanistic studies revealed that the anti-inflammatory properties of FLZ are mediated through inhibition of nicotinamide adenine dinucleotide phosphate oxidase (NADPH oxidase, also called PHOX), the key microglial superoxide-producing enzyme. Finally, animal studies showed that FLZ significantly protects against 1-methyl-4-phenyl-1, 2, 3, 6-tetrahydropyridine (MPTP)-induced DA neuronal loss, microglia activation and behavioral changes.

## Methods

### Animals and treatment

Timed-pregnant Fisher F344 rats were obtained from Charles River Laboratories (Raleigh, NC). Eight-week-old male C57BL/6J mice, weighing 25–28 g, were maintained on a 12:12 h light:dark cycle and fed ad libitum. PHOX-deficient (gp91 PHOX^-/-^) and wild-type C57BL/6J (gp91 PHOX^+/+^) mice were obtained from the Jackson Laboratory (Bar Harbor, ME). Animals were adapted for 2 weeks to the conditions described above before experimentation. To examine the effect of FLZ on MPTP-induced neurotoxicity, mice received daily MPTP injections [15 mg/kg of MPTP.HCl, s.c.] for 6 consecutive days. From the third day on, FLZ (75 mg/kg, p.o.) was administered 30 min before every MPTP injection for the last 4 days. Housing, breeding, and experimental use of the animals were performed in strict accordance with the National Institutes of Health guidelines.

### Reagents

FLZ was kindly provided by Professor Xiaotian Liang in the Department of Pharmaceutical Chemistry, Institute of Materia Medica, Chinese Academy of Medical Science. It is a white powder with 99% purity. The polyclonal anti-tyrosine hydroxylase (TH) antibody was a generous gift from Dr. John Reinhard (GlaxoSmithKline, Research Triangle Park, NC). LPS (strain O111:B4) and 2', 7'-dichlorofluorescin diacetate were purchased from Calbiochem (San Diego, CA). [^3^H]-DA (28 Ci/mmol) was purchased from PerkinElmer Life Sciences Inc. (Boston, MA). The mouse monoclonal antibody raised against OX42 antigen was purchased from Chemicon International (Temecula, CA). Rabbit anti-p47^*phox *^was obtained from Millipore Corporation (Bedford MA). FITC-conjugated goat anti-rabbit IgG antibody was obtained from Jackson ImmunoResearch Laboratories Inc. (West Grove, PA). Rabbit anti-GAPDH antibody was obtained from Abcam (Cambridge, MA). Mouse anti-gp91^*phox *^antibody was purchased from BD Transduction Laboratories (San Jose, CA). All other reagents came from Sigma (St Louis, MO).

### Cell cultures

Primary rat mesencephalic neuronal-glial cultures were prepared as described previously [18]. Briefly, after the ventral mesencephalic tissues were removed and dissociated by a mechanical triturating, cells were seeded at 5 × 10^5^/well to 24-well culture plates or 1.5 × 10^5^/well to 96-well culture plates in maintenance medium. Seven-day-old cultures were used for treatment. Immunocytochemical analysis indicated that at the time of treatment the cultures were made up of ~10% microglia, ~50% astroglia, and ~40% neurons, of which 1 ~2% were TH-immunoreactive neurons.

Primary microglia-enriched cultures were prepared from the whole brains of 1 or 2-day-old mice, as described previously [19]. Briefly, brain tissues, devoid of meninges and blood vessels, were dissociated by mechanical trituration. The isolated cells (5 × 10^7^) were seeded in 150 cm^2 ^culture flasks in Dulbecco's modified Eagle medium. Upon reaching confluence (12–14 days), microglia were separated from astroglia by shaking the flasks at 180 rpm for 1 h. Purity of the microglia-enriched cultures was >98%, as determined by immunocytochemical staining.

The rat microglia HAPI cells were generous gifts from Dr. James R. Connor (Pennsylvania State University, Hershey, PA). They were maintained at 37°C in Dulbecco's modified Eagle's medium supplemented with 10% fetal bovine serum, 50 U/ml penicillin, and 50 μg/ml streptomycin in a humidified incubator with 5% CO_2_/95% air.

### DA uptake assay

Degeneration of DA neurons was assessed by measuring the ability of cultures to take up DA. DA uptake assays were performed as previously described [18]. Briefly, after two washes with warm Krebs-Ringer buffer the cultures were incubated for 20 min at 37°C with 1 μM [^3^H]-DA for DA uptake. Then the cultures were washed 3 times with ice-cold KRB, and the cells were then dissolved in 1 N NaOH. Radioactivity was determined by liquid scintillation counting. Nonspecific DA uptake, determined in the presence of mazindol (10 μM), was subtracted.

### Immunocytochemistry

DA neurons were recognized with an anti-TH antibody, and microglia were detected with the OX-42 antibody, as described previously. For morphological analysis, images were acquired using an inverted microscope (Nikon, Tokyo, Japan) connected to a camera (DAGE-MTI, Michigan City, IN) operated with the MetaMorph software (Universal Imaging Corporation, Downingtown, PA). To quantify cell numbers, total TH positive cells in entire wells were counted by three individuals. The average of these scores was reported.

### Assay for inflammatory factors

For the TNF-α assay, culture supernatant was collected after 3 h of stimulation with LPS. The concentration of TNF-α was measured with a mouse TNF-α enzyme-linked immunosorbent assay kit from Genzyme (Cambridge, MA).

The production of NO was assessed as the accumulation of nitrite in the culture supernatant, using a colorimetric reaction with the Griess reagent. The culture supernatants were collected after 24 h of stimulation with LPS and mixed with equal volumes of the Griess reagent. The absorbance at wavelength 540 nm was measured with a UV MAX kinetic microplate reader (Molecular Devices).

The production of PGE_2 _in the enriched microglial cultures was evaluated 24 h after LPS treatment using a PGE_2 _EIA kit from Cayman (Ann Arbor, MI), according to the manufacturer's instructions.

### Assay for reactive oxygen species (ROS)

Production of superoxide was determined by measuring the superoxide dismutase (SOD)-inhibitable reduction of the water-soluble tetrazolium salt (WST-1) 30 min after LPS treatment [20]. Microglia-enriched cultures in 96-well culture plates were washed twice with Hanks' Balanced Salt Solution (HBSS) without phenol red. Cultures were then incubated at 37°C for 1 h with vehicle control or FLZ in HBSS (50 μl/well). Then, 50 μl of HBSS with and without SOD (50 U/ml) was added to each well along with 50 μl of WST-1 (1 mM) in HBSS and 50 μl of vehicle or LPS (2 ng/ml). Thirty minutes later, the absorbance at 450 nm was read with a SpectraMax Plus microplate spectrophotometer (Molecular Devices, Sunnyvale, CA).

The production of intracellular ROS was measured by 2', 7'-dichlorofluorescin diacetate oxidation 2 h after LPS treatment. Microglia-enriched cultures were seeded (5 × 10^4^) in 96-well plates and then exposed to 20 μM 2', 7'-dichlorofluorescin diacetate for 1 h, followed by pretreatment with FLZ for 1 h and treatment with HBSS containing LPS. After incubation, the fluorescence was read at the 485 nm excitation and 530 nm emission on a fluorescence plate reader. Cell-free experiments with and without FLZ were conducted to determine that the reagents themselves did not alter fluorescence.

### Real-time RT-PCR analysis

The primers synthesized from Sigma Genosys were used as follows: TNF-α, forward (TCGTAGCAAACCACCAAGCA) and reverse (CCCTTGAAGAGAACCTGGGAGTA); iNOS, forward (GTGCTAATGCGGAAGGTCATG) and reverse (CGCTTCCGACTTTCCTGTCT); COX_2_, forward (CCAGCAGGCTCATACTGATAGGA) and reverse (GCAGGTCTGGGTCGAACTTG); GAPDH, forward (CCTGGAGAAACCTGCCAAGTAT) and reverse (AGCCCAGGATGCCCTTTAGT); and gp91, forward (CCTGCAGCCTGCCTGAATT) and reverse (AAGGAGAGGAGATTCCGACACA).

### Flow cytometry

HAPI cells were treated with 10 ng/ml LPS for 24 h in the presence or absence of 10 μM FLZ. The cells were removed from culture and washed twice with cold FACS buffer (saline with 5% normal goat serum). Cells were incubated with FcR block for 30 min at 4°C and then incubated for 45 min at 4°C with PE-conjugated OX6 antibody or the appropriate isotype control antibody (BD Pharmingen). After antibody binding, cells were washed and fixed. Fluorescence was analyzed on a FACSCalibur (BD Biosciences). The mean fluorescent intensity (MFI) of experimental group was determined by subtracting the MFI of the isotype control from the MFI of the unstimulated or LPS-stimulated microglia.

### Subcellular fractionation and western blot analysis

Subcellular fractionation was performed as described previously [21]. HAPI cells, seeded in dishes at 5 × 10^4 ^cells/well, were treated with 10 ng/ml LPS for 10 min in the absence or presence of 10 μM FLZ for 1 h. The cells were then lysed in hypotonic lysis buffer, incubated on ice for 30 min, and subjected to Dounce homogenization (~20–25 strokes, tight pestle A). The lysates were loaded onto sucrose in lysis buffer and centrifuged at 1,600 *g *for 15 min; the supernatant above the sucrose gradient was used as the cytosolic fraction after centrifugation at 150,000 *g *for 30 min. The pellets, solubilized in 1% Nonidet P-40 hypotonic lysis buffer, were used as the membranous fraction. For western blot analysis, the primary antibodies included anti-p47^*phox *^(1: 2000), anti-GAPDH (1:2000) and anti-gp91^*phox *^(1:2000).

### Rota-rod measurement

Quantitative measurements of motor coordination in mice were performed using an accelerating rota-rod (model 7650, UGO Basile, Comerio, VA, Italy). The rota-rod consisted of a plastic rod (diameter = 3 cm; length = 30 cm) partitioned off with round plates to prevent the mice from escaping from the sides of the rod. The rod was covered with smooth plastic tubing and suspended 16 cm above five plastic levers attached to timers that stop when mice land on the lever surface. The mice were placed on the rod that rotated at 3 rpm at 0 min and accelerated from 4 to 20 rpm over 5 min. Mice were oriented perpendicular to the long axis of the rod, such that the mice had to make forward walking movements to avoid falling. The latency time to fall was measured using mice 28 days after their first dose of MPTP, with each mouse experiencing three trials per day with an intertrial interval of 10 min. The average latency of each mouse was calculated.

### Immunohistochemistry and cell counting in the SNpc of C57BL/6 mice

Mouse brains were cut on a horizontal sliding microtome into transverse free-floating sections, 35 μm in thickness. Six to eight brain sections of SNpc were collected at intervals of 140 μm. For immunohistochemistry, the following concentrations of antibodies were used: anti-TH (1:5000), anti-Iba-1 (1: 1000), biotinylated anti-mouse IgG (1:1000). Digital images of TH neurons in SNpc were acquired on an Olympus microscope (Olympus^®^, Tokyo, Japan) using an attached Polaroid digital microscope camera (Polaroid^®^, Cambridge, MA, USA). Twenty four consecutive brain slices (35-μm thickness), which encompassed the entire substantia nigra compacta, were collected. A normal distribution of the number of TH-IR neurons in the SNpc was constructed based on the counts of 24 slices from saline-treated mice, MPTP-treated mice and FLZ-treated mice. The distribution curves from these groups superimpose and show no difference in number and shape of the curves. Six to eight evenly spaced brain slices from different groups of animals were immunoreacted using an antibody against TH, and immunoreactive cells were counted. The distribution of cell numbers from each animal was matched with the normal distribution curve to correct for errors resulting from the cutting. Three individuals performed counting in a double-blind manner. Conclusions were drawn only when the interobserver differences were within 5%. A mean value for the number of SNpc TH neurons was then deduced by averaging the counts of 6–8 sections for each animal.

### Statistical analysis

The data are presented as mean ± S.E.M. For multiple comparisons of groups, two-way ANOVA was used. Statistical significance of differences between groups was assessed using paired Student's t test, followed by Bonferroni correction using the JMP program (SAS Institute, Cary, NC, USA). A value of P < 0.05 was considered statistically significant.

## Results

### FLZ protects DA neurons against LPS-induced degeneration

**Figure 2:**
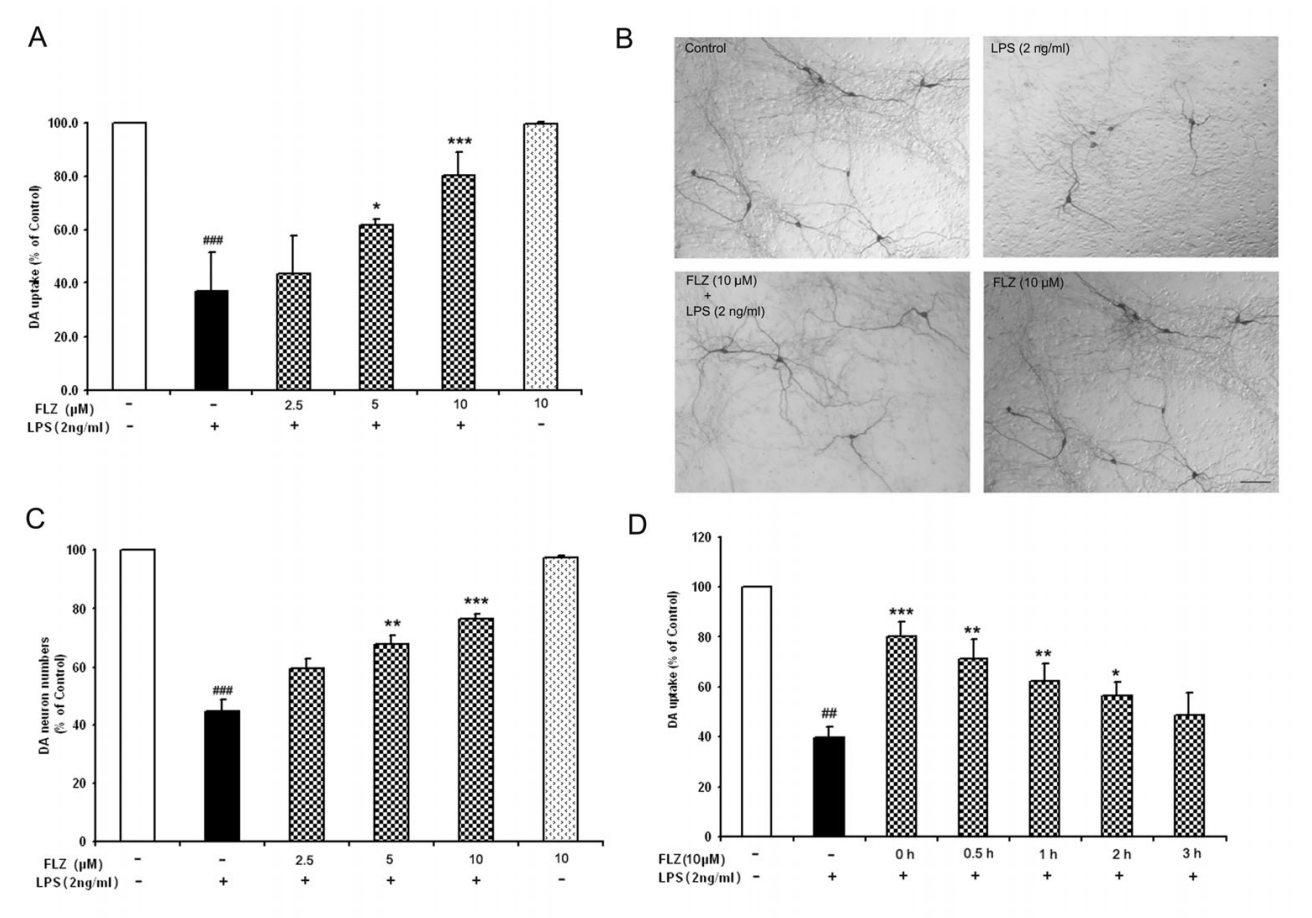
**FLZ functionally and morphologically protects DA neurons from LPS-induced neurotoxicity in rat mid-brain neuron-glia culture**. Neuronal-glial cultures were pre-treated with different concentrations of FLZ for 1 h followed by 2 ng/ml LPS; 7 days later, DA neurotoxicity was measured by [^3^H]-DA uptake assay **(A) **and by immunocytochemical analysis. Representative pictures of immunoreactions are shown in **(B) **and TH neuron counts are shown in **(C)**. For assessment of FLZ treatment following neurotoxic insult, neuronal-glial cultures were first treated with 2 ng/ml LPS. Then, 0, 0.5, 1, 2, and 3 h later, 10 μM FLZ was added to the cultures. DA neurotoxicity was measured by [^3^H]-DA uptake assay 7 days later **(D)**. The data are expressed as percentages of control culture values, and represent the mean ± S.E.M. for three independent experiments, each performed with triplicate samples. ## *p *< 0.01 and ### *p *< 0.001 compared with vehicle-treated control cultures; * *p *< 0.05, ** *p *< 0.01 and *** *p *< 0.001 compared to LPS treatment group. Scale bar, 100 μm.

Mesencephalic neuronal-glial cultures were pretreated with FLZ or vehicle for 1 h, or post-treated with 10 μM FLZ at 0, 0.5, 1, 2 and 3 h after LPS treatment. Seven days later, the degeneration of DA neurons was assessed. [^3^H]DA uptake assays indicated that LPS treatment reduced the uptake capacity to about 40% of that of vehicle-treated control cultures (Fig. 2A). FLZ attenuated LPS-induced DA uptake decreases in a dose-dependent manner; whereas 10 μM FLZ alone did not affect DA uptake in cultures. Morphologically, in addition to reductions in the abundance of TH neurons, the dendrites of the remaining TH neurons in LPS-treated cultures were significantly less elaborate than those in the control group (Fig. 2B). FLZ significantly attenuated these morphological changes in TH neurons induced by LPS. These protective effects of FLZ were confirmed by the cell count numbers of TH neurons (Fig. 2C). For the post-treatment groups, 10 μM FLZ showed a protective effect even when given 2 h after LPS treatment (Fig. 2D). However, when 10 μM FLZ was added at 3 h post-LPS treatment, no significant protective effect was observed.

### FLZ's neuroprotective effect against LPS-induced DA neurotoxicity is mediated through microglia

**Figure 3:**
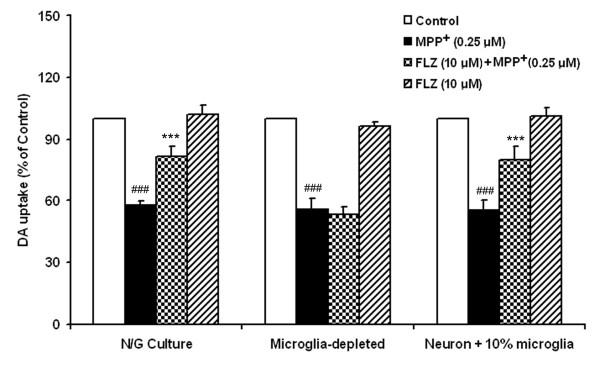
**Microglia (but not astroglia) mediate the neuroprotection of FLZ**. Different kinds of cultures were pretreated with 10 μM FLZ for 1 h, and then 0.25 μM MPP^+ ^was added. DA neurotoxicity was measured by [^3^H]-DA uptake assay 7 days later. The data are expressed as percentages of control culture values, and represent the mean ± S.E.M. for three independent experiments, each performed with triplicate samples. ### *p *< 0.001 compared with vehicle-treated control cultures; *** *p *< 0.001 compared to MPP^+ ^treatment group.

In addition to neurons, the mesencephalic neuron-glia cultures also contain ~10% microglia and ~50% astrocytes. To evaluate the potential contribution of glia in mediating the neuroprotective effect of FLZ, we performed cell reconstitution experiments. As shown in Fig 3, 1-methyl-4-phenylpyridine (MPP^+^) reduced DA uptake by about 52% in neuronal-glial cultures, but 10 μM FLZ significantly protected neurons against this damage. However, 10 μM FLZ failed to show any protective effect against the toxicity of MPP^+ ^in microglia-depleted cultures, which contain only neurons and astrocytes. In contrast, when 10% microglia were added back to the neuron-enriched cultures, the MPP^+^-induced reduction in DA uptake could be reversed by 10 μM FLZ. These results demonstrate that microglia, but not astroglia, mediate FLZ's neuroprotective effect against LPS-induced neurodegeneration.

### FLZ suppresses LPS-induced microglia activation

**Figure 4:**
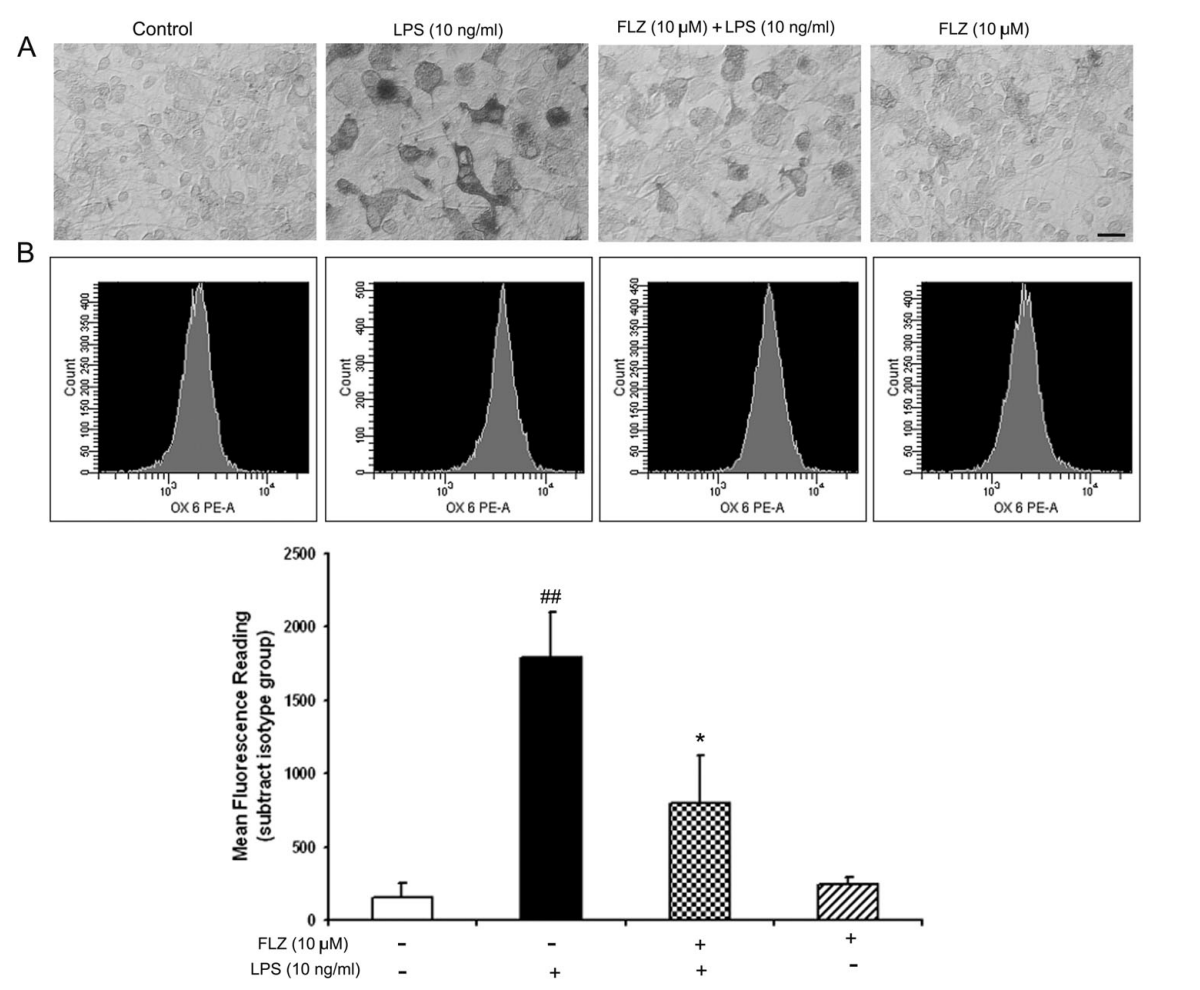
**FLZ attenuates LPS-induced microglia activation**. **(A)**: Neuronal-glial cultures were pretreated with 10 μM FLZ for 1 h followed by 2 ng/ml LPS; 24 h later, activation of microglia was assessed by OX-42 immunohistochemistry. Representative results are illustrated. **(B)**: HAPI microglia cells were pretreated with 10 μM FLZ for 1 h followed by stimulation with 10 ng/ml LPS for 24 h. Expression of MHC class II antigen was detected by flow cytometry. The cells were analyzed on a FACS Calibur, and the MFI of experimental groups was determined by subtracting the MFI of the isotype control from the MFI of each group. The data are expressed as mean ± S.E.M. for three independent experiments, each performed with triplicate samples. ## *p *< 0.01 compared with vehicle-treated control group; * *p *< 0.05 compared to LPS treatment group. Scale bar, 50 μm.

Immunohistochemical studies using the OX-42 antibody, a marker for rat microglia, showed LPS-induced morphological evidence of microglial activation in mesencephalic neuronal-glial cultures. LPS treatment transformed microglia from a predominantly resting, round, small cell morphology to an activated, rod- and/or amoeboid-shaped cell morphology with intensified immunoreaction (Fig. 4A). LPS-stimulated activation of microglia was partially suppressed by pretreating the cultures with 10 μM FLZ, whereas the same concentration of FLZ alone did not show significant effects on microglial morphology. The effect of FLZ on activated microglia was further confirmed by flow cytometric analysis using an anti-MHC-II antibody, a marker for the activated microglia. Quantitative analysis revealed that the degree of 10 ng/ml LPS-induced activation of microglia was decreased by 60% by pretreatment with 10 μM FLZ (Fig. 4B). Rat HAPI-immortalized microglial cells were used for this flow cytometric analysis because most of the primary microglial cells died during the experimental procedure.

### FLZ suppresses LPS-induced release of proinflammatory mediators and their gene expression

**Figure 5:**
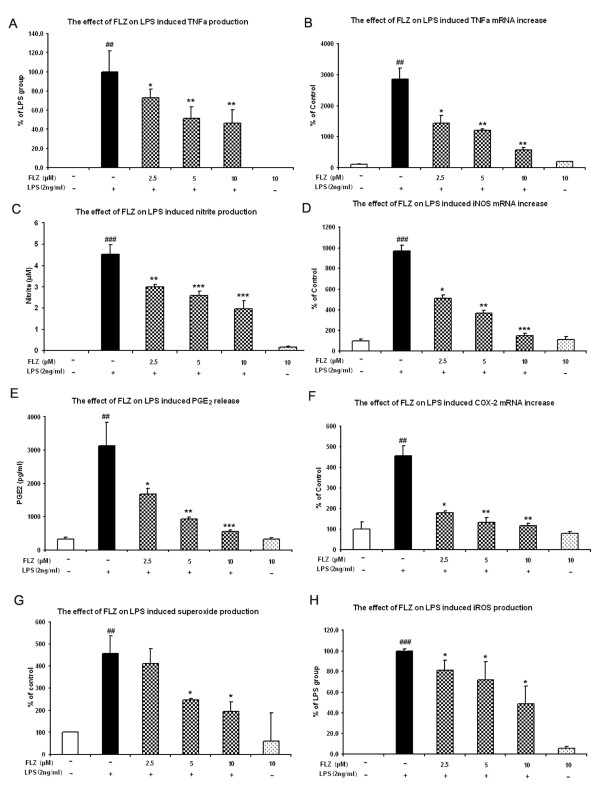
**FLZ inhibites LPS-induced production of proinflammatory factors and their gene expression in microglia**. The effects of FLZ on LPS-induced production of TNF-α, NO, and PGE_2 _(**A**, **C**, **E**, respectively) or TNF-α, iNOS, and COX-2 mRNA expression (**B**, **D**, **F**, respectively) and the production of superoxide (**G**), and intracellular ROS (**H**) are shown. Enriched microglia cells were treated with different concentrations of FLZ or with vehicle for 1 h before addition of 2 ng/ml LPS. For mRNA analysis, total RNA was harvested 3 h after LPS treatment, followed by real-time RT-PCR analysis of iNOS, TNF-α, COX-2 and GAPDH using specific primers. The data are expressed as mean ± S.E.M. for three independent experiments, each performed with triplicate samples. ## *p *< 0.01 and ### *p *< 0.001 compared with vehicle-treated control cultures; * *p *< 0.05, ** *p *< 0.01 and *** *p *< 0.001 compared to LPS treatment group.

Accumulating evidence indicate that proinflammatory factors released by over-activated microglia in dysregulated inflammatory conditions play a key role in DA neurodegeneration [18,19,22]. Therefore, we hypothesized that FLZ would dampen LPS-stimulated production of proinflammatory factors in microglia-enriched cultures. We found that pretreatment with FLZ at concentrations ranging from 2.5 to 10 μM significantly blocked LPS-stimulated production of proinflammatory mediators and their mRNA expression in a dose-dependent manner. These gene products include TNF-α (Fig. 5A and 5B), inducible nitric oxide synthase (iNOS) (Fig. 5C and 5D), and COX-2 (Fig. 5E and 5F). In addition, FLZ significantly attenuated LPS-induced ROS production including extracellular superoxide (Fig. 5G) and intracellular ROS (Fig. 5H).

### FLZ protects against LPS-induced neuronal degeneration through inhibition of PHOX activation

**Figure 6:**
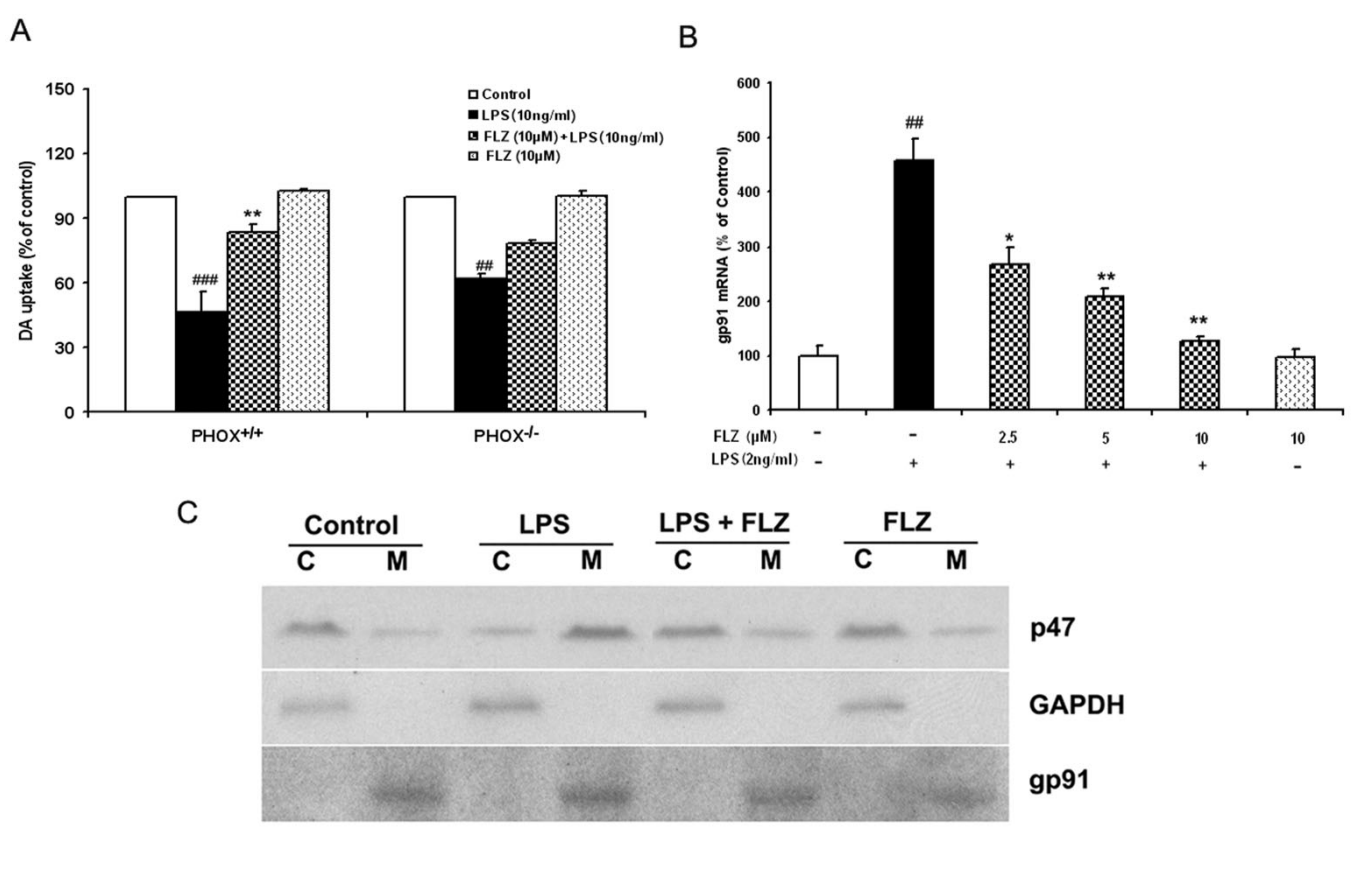
**Microglial PHOX is the target of FLZ's inhibition in LPS-induced neurotoxicity**. **(A)**: The role of PHOX in FLZ's protective effect. PHOX^+/+ ^and PHOX^-/- ^mouse neuronal-glial cultures were pretreated with vehicle or with 10 μM FLZ for 1 h, followed by 10 ng/ml LPS treatment. Neurotoxicity was assessed by [^3^H]-DA uptake 7 days later. **(B)**: The effect of FLZ on LPS-induced gp91 mRNA expression. Enriched microglia cells were treated with vehicle or with different concentrations of FLZ for 1 h before the addition of 2 ng/ml LPS. For mRNA analysis, total RNA was harvested 3 h after LPS treatment, followed by real-time reverse transcription-PCR analysis using specific primers. **(C)**: Effect of FLZ on cytosolic p47^*phox *^protein translocation. HAPI cells were pretreated with vehicle or with 10 μM FLZ for 1 h followed by 10 ng/ml LPS treatment for 10 min. Subcellular fractions were isolated to perform western blot analysis. C = cytosolic extract; M = membrane extract. GAPDH and gp91^*phox *^are used as internal cytosolic and membrane controls, respectively. The data are expressed as percentages of control culture values and represent the mean ± S.E.M. for three independent experiments, each performed with triplicate samples. ## *p *< 0.01 and ### *p *< 0.001 compared with vehicle-treated control group; * *p *< 0.05 and ** *p *< 0.01 compared to LPS treatment group.

Among the proinflammatory factors released from over-activated microglia, production of superoxide from PHOX has been shown to exert direct neurotoxicity and to regulate gene expression for other proinflammatory factors [19]. To further investigate the role of ROS in FLZ-elicited neuroprotective effects, we used neuronal-glial cultures from mice deficient in the catalytic subunit (gp91^PHOX^) of PHOX, which is the key enzyme required for the production of ROS. As shown in Fig. 6A, LPS treatment of neuronal-glial cultures prepared from PHOX^+/+ ^mice substantially reduced [^3^H]-DA uptake and, similarly, FLZ attenuated this decrease. In contrast, LPS treatment also showed a significant but smaller reduction in [^3^H]-DA uptake capacity in cultures from PHOX^-/- ^mice, while FLZ's protective effect was reduced significantly. Further, results from real-time PCR showed that FLZ inhibited the gp91 mRNA expression induced by LPS treatment (Fig. 6B). Therefore, our data support the idea that PHOX plays an important role in FLZ-mediated protection against LPS-induced neuronal degeneration.

It has been reported that activation of PHOX holoenzyme is required to initiate the translocation of its cytosolic components p47^*phox *^into the plasma membrane [23]. Because of the potent inhibition of ROS production by FLZ, we sought to determine whether FLZ inhibits PHOX activation by preventing this translocation. Data from subcellular fractionation and western blot analysis clearly showed an increase in immunoreactivity of p47^*phox *^in the membrane of microglia 10 min after LPS treatment (Fig. 6C), and this increase in immunoreactivity was significantly blocked in the presence of FLZ. Thus, this result further proves that FLZ-mediated inhibition of superoxide production by LPS is mediated through suppression of p47^*phox *^membrane translocation; that is, activation of PHOX.

### FLZ inhibits MPTP-induced DA neurotoxicity, microglial activation and motor function deficiency in mice

To further substantiate the neuroprotective effect of FLZ shown in these cell culture studies, we performed an animal study with daily injections of MPTP for 6 days.

**Figure 7:**
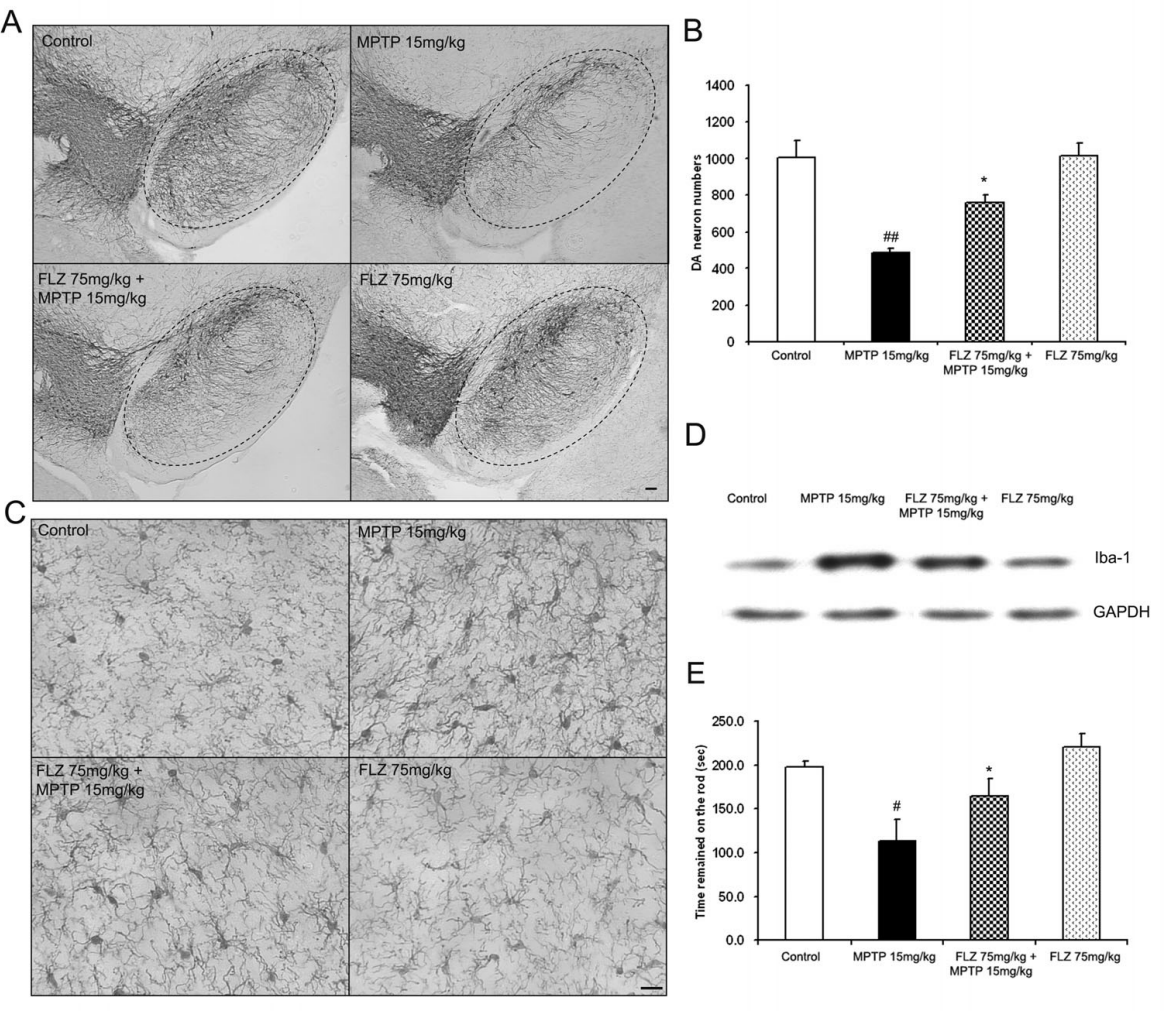
**FLZ shows significant protective effect against the toxicity of MPTP *in vivo***. Eight-week-old male C57BL/6J mice received daily MPTP injections (15 mg/kg, s.c.) for 6 consecutive days. From the third day on, FLZ (75 mg/kg, p.o.) was administered 30 min before every MPTP injection for the last 4 days. DA neurotoxicity was measured by immunohistochemistry using an anti-TH antibody **(A) **and cell counting of DA neurons **(B) **in the SNpc of different groups of mice. To assess activation of microglia, SNpc brain sections were stained using Iba-1 antibody, a microglial maker **(C)**. To obtain quantitative data, the midbrains of the mice were dissected out, and levels of Iba-1 in these midbrains were determined using western blot assays **(D)**. To assess the protective effect of FLZ on MPTP-induced motor function deficiency, a rotor-rod assay was used and the time for each mouse to remain on the spinning rod was recorded **(E)**. The data are expressed as mean ± S.E.M. for the results of 6–8 mice. # *p *< 0.05 and ## *p *< 0.01 compared with vehicle-treated control group; * *p *< 0.05 compared to MPTP treatment group. Scale bar, 50 μm.

Immunohistochemical analysis (Fig. 7A) revealed that MPTP injection caused significant DA neuronal loss in SNpc. Both number of DA neurons and extent of neuronal processes were significantly lost in MPTP-treated group mice. For a second group, FLZ was given to the mice starting on the third day of MPTP treatment and injections of FLZ were continued daily for the next four days. This post-treatment regimen with FLZ significantly reduced MPTP-induced DA neuronal loss. Cell count analysis showed that MPTP treatment decreased the number of DA neurons in the SNpc to about 45% of the vehicle treated group, while post-treatment with FLZ increased the number of remaining neurons to about 75% of the vehicle-treated group. For a third group of mice that received only FLZ, the number of DA neurons did not differ from the vehicle-treated group (Fig. 7B). Morphological changes of microglia revealed by immunoreaction with Iba-1 antibody were used as an index for inflammatory conditions. MPTP treatment transformed most of the microglia from resting cells to activated, large cells, and this activation of microglia was significantly suppressed by FLZ post-treatment (Fig. 7C). To obtain quantitative data, the midbrains of the mice were dissected out and changes in Iba-1 immunoreactivity were determined using western blot assays. The results confirmed that FLZ suppressed MPTP-induced microglial activation (Fig. 7D). To access the locomotor activities of the mice, the rota-rod test was used. At 28 days after the first MPTP injection, the length of time that MPTP-group mice remained on the rod was much less than that of vehicle-group mice, while post-treatment with FLZ significantly prolonged the time that the mice were able to remain on the rod (Fig. 7E). These results from rota-rod testing thus corroborated the immunohistochemical results showing loss of DA neurons.

## Discussion

In this study, we demonstrate that FLZ significantly reduces LPS-induced degeneration of DA neurons in primary mesencephalic neuronal-glial cultures, as evidenced by DA uptake and TH immunoreaction of DA neurons. Furthermore, post-treatment with FLZ, 2 h after LPS challenge, was still effective in providing neuroprotection. Mechanistic studies revealed that inhibition of microglial PHOX activation by FLZ underlay the neuroprotective effect of FLZ. Reduction of PHOX-generated superoxide was a critical initial site of action of FLZ, which in turn resulted in suppression of a wide array of proinflammatory mediators. In animal studies, FLZ significantly ameliorated MPTP-induced DA neuronal loss in SNc and motor function deficits even when administered 2 days after MPTP intoxication.

In the brain, two types of glial cells, astroglia and microglia, are the main players in the neuroinflammatory process [24]. Astroglia serve to maintain homeostasis and secrete neurotrophic factors that promote neuronal survival. Microglia, on the other hand, play a role in immune surveillance under normal conditions, and become readily activated in response to infections and neuronal injuries under pathological conditions [25]. Microglial pro-inflammatory factors such as NO, TNF-α, PGE2 and ROS serve immune surveillance functions by killing foreign microorganisms [26]. However, over-activation of microglia results in overproduction of proinflammatory factors, which in turn results in neuronal death in the brain [27-29]. It is generally accepted that neuroinflammation generated by dysregulated microglia plays a critical role in neurodegenerative disorders, such as PD. We and others have documented that microglial proinflammatory and neurotoxic factors such as free radicals, cytokines, chemokines, and prostaglandins may damage surrounding neurons and cause neurodegeneration [19,22,30,31]. Hence, agents that can inhibit the production of proinflammatory and neurotoxic factors may be highly desirable candidates for the development of potential therapeutic agents. In this study, we show that FLZ significantly inhibits microglial activation and microglial production of proinflammatory factors, as well as the generation of free radicals such as superoxide anion and iROS, and we show corresponding changes at both the mRNA and protein levels. This study provides strong evidence to indicate that the inhibitory effect of FLZ on the production of these factors is a major mechanism in its significant protection of DA neurons against inflammation-mediated degeneration.

Among the various factors released from microglia, superoxide generated by PHOX plays the most critical role in the subsequent neuroinflammatory processes and neurodegeneration by producing direct damage to DA neurons and promoting the production of other proinflammatory factors [31-33]. Activation of PHOX in microglia not only increases the production of superoxide but indirectly increases intracellular ROS concentrations, possibly through conversion of superoxide to H_2_O_2_, which is membrane permeable. Increases in intracellular ROS can enhance activation of NF-κB, which would lead to higher TNF-α and PGE_2 _production [19,26]. In addition, it has been reported that PHOX inhibitors prevent LPS/IFNγ-induced degradation of IκBα, and thus inhibit activation of NF-κB [34]. Thus, PHOX is an ideal target for developing anti-inflammatory drugs due to its dual roles in inflammation-related neuronal damage, and due to its high degree of cell-type specificity in immune cells such as microglia, macrophages, and neutrophils [19,22].

The fact that FLZ significantly inhibits the production of superoxide induced by LPS led us to examine this notion in greater detail by using PHOX^-/-^deficient mutant mice. The finding that FLZ could significantly lessen LPS-induced DA uptake reduction in cells from wild-type mice, but its protective effect is less significant in cultures from PHOX^-/- ^mice, strongly support the contention that the protective effect of FLZ is probably mediated through inhibition of PHOX activity.

Since these results pointed to the importance of FLZ/PHOX interactions, we further studied the molecular mechanism underlying inhibition of PHOX by FLZ. PHOX is a multicomponent complex, dormant in non-activated cells, that catalyzes during phagocytosis and increases the production of superoxide anion O_2_^-^. The oxidase is composed of a membrane-bound heterodimeric flavocytochrome, consisting of two subunits, gp91^phox ^and p22^phox^, four cytosolic subunits, p47^phox^, p67^phox^, p40^phox^, and the small GTPase Rac. All of the redox stations of the oxidase are located on gp91^phox ^and, in the resting state, are not engaged in electron transport. O_2_^- ^production is, most likely, the result of a conformational change in gp91^phox^, consequent to the translocation of cytosolic components to the membrane environment, and the interaction of one or more components with cytochrome *b*_559_, a process termed oxidase assembly [35,36]. Western blot analysis from this study revealed that FLZ inhibited LPS-induced p47^phox ^translocation from cytosol to membrane. Thus, FLZ inhibited the activation of PHOX by inhibiting its assembly, thereby inhibiting the activation of microglia and the production of various proinflammatory factors and subsequently resulting in a neuroprotective effect.

The neuroprotective effect of FLZ shown in our cell culture studies was further confirmed in animal studies using MPTP as a neurotoxin. Reactive microgliosis after MPTP treatment has been documented by our laboratory and others [33,37]. It is believed that the initial damage to or death of DA neurons caused by MPTP could signal the activation of microglia through either the release of soluble factors from damaged neurons or the loss of cell-cell contact inhibition between neurons and microglia [30,38,39]. Reactive microgliosis may release additional proinflammatory and neurotoxic factors to augment the neuroinflammatory process and cause further damage to the remaining DA neurons. Thus, the continuing processes involving death of DA neurons, secondary activation of microglia (reactive microgliosis), and further neuronal damage would create a vicious "self-propelling" circle and trigger progressive neurodegeneration [15,26]. We find that FLZ significantly prevents DA neuronal loss in SNpc after MPTP treatment. The efficacy of neuroprotection correlates well with amelioration of behavioral locomotor deficiency. The anti-inflammatory effect of FLZ in the MPTP model was due to reduced reactive microgliosis elicited after MPTP treatment as evidenced by immunohistochemical analysis of activated microglia. The effectiveness of FLZ in this animal study strongly confirms the protective effect of FLZ, and the fact that this effect was evident when FLZ was given 2 days after MPTP challenge is an important consideration for the potential of FLZ for clinical use in PD patients. Further, we measured MPP^+ ^concentrations in the brains of mice from each group, and found that FLZ didn't affect these MPP^+ ^concentrations (data not shown).

## Conclusion

In summary, this study showed potent efficacy of FLZ in protecting DA neurons against the neurotoxicity induced by LPS and MPTP. This protective effect was related to FLZ's effect in preventing over-activation of microglia. FLZ inhibition of microglial activation and of ROS production by inhibiting PHOX assembly underlay the FLZ-elicited suppression of inflammatory factor production and release by microglia. These findings, combined with the fact that FLZ is a small molecule that can be taken orally suggest that FLZ has potential as a new therapeutic compound for treatment and prevention of chronic neurodegenerative diseases such as PD.

## Competing interests

The authors declare that they have no competing interests.

## Authors' contributions

DZ performed majority of the experiments and drafted the manuscript, XMH, SJY, JL, HMG and LQ participated in the experiments, BW carried out the immunohistochemical assays, GTL and JSH conceived the study and its design and helped to draft the manuscript. All authors read and approved the final manuscript.
